# The genome sequence of knotweed leaf beetle, *Gastrophysa polygoni* (Linnaeus, 1758)

**DOI:** 10.12688/wellcomeopenres.23293.1

**Published:** 2024-11-04

**Authors:** James McCulloch, University of Oxford and Wytham Woods Genome Acquisition Lab, Darwin Tree of Life Barcoding collective, Wellcome Sanger Institute Tree of Life Management, Samples and Laboratory team, Wellcome Sanger Institute Scientific Operations: Sequencing Operations, Wellcome Sanger Institute Tree of Life Core Informatics team, Tree of Life Core Informatics collective, Darwin Tree of Life Consortium

**Affiliations:** 1 University of Oxford, Oxford, England, UK; 2 Tree of Life, Wellcome Sanger Institute, Hinxton, England, UK

**Keywords:** Gastrophysa polygoni, knotweed leaf beetle, genome sequence, chromosomal, Coleoptera

## Abstract

We present a genome assembly from a female specimen of *Gastrophysa polygoni* (knotweed leaf beetle; Arthropoda; Insecta; Coleoptera; Chrysomelidae). The genome sequence has a total length of 369.80 megabases. Most of the assembly (99.89%) is scaffolded into 12 chromosomal pseudomolecules, including the X sex chromosome. The mitochondrial genome has also been assembled and is 17.11 kilobases in length.

## Species taxonomy

Eukaryota; Opisthokonta; Metazoa; Eumetazoa; Bilateria; Protostomia; Ecdysozoa; Panarthropoda; Arthropoda; Mandibulata; Pancrustacea; Hexapoda; Insecta; Dicondylia; Pterygota; Neoptera; Endopterygota; Coleoptera; Polyphaga; Cucujiformia; Chrysomeloidea; Chrysomelidae; Chrysomelinae; Chrysomelini; *Gastrophysa*; *Gastrophysa polygoni* (Linnaeus, 1758) (NCBI:txid878018).

## Background

**Figure 1.**
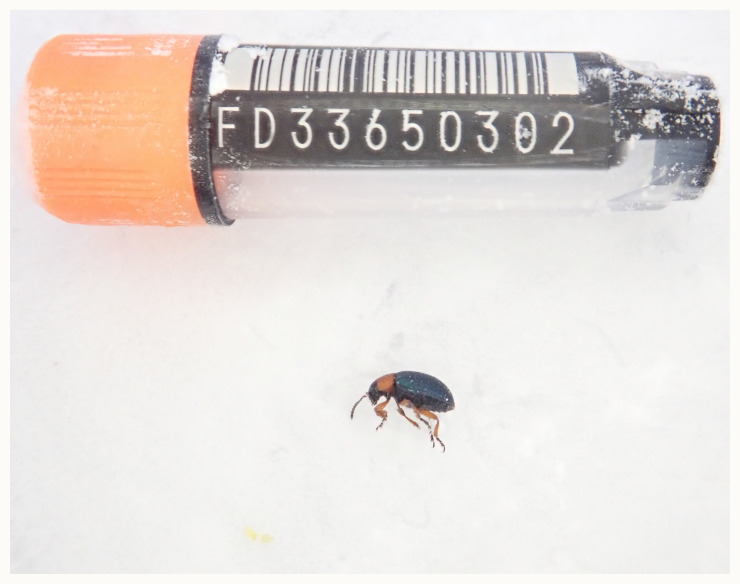
Photograph of the *Gastrophysa polygoni* (icGasPoly1) specimen used for genome sequencing.

*Gastrophysa polygoni* ( Figure 1) is a strikingly coloured beetle in the family Chrysomelidae, commonly known as the leaf beetles. This species is decorated with a pair of metallic blue-green elytra contrasting with a reddish-orange pronotum. This colouration easily distinguishes it from its sole British congener, *G. viridula*, which is entirely metallic green ( Duff, 2016). The genus *Gastrophysa* has two further species which are found outside of the UK, both in a separate subgenus ( *Exiguipenna*) to the British species ( *Gastrophysa sensu stricto*) and distributed elsewhere in Europe.

*G. polygoni* is widespread in the United Kingdom, but becomes more sparse further north, with the northernmost record displayed on the NBN Atlas being just north of Inverness ( NBN Atlas Partnership, 2024). Outside of the UK, this species’ native distribution stretches from western Europe to China and Siberia. It has expanded its range as a non-native species in North America, where it can be found across Canada from the Pacific to the Atlantic coast, as well as in the United States at least as far south as Montana ( Lesage & Majka, 2009).

The significance of *G. polygoni* predominantly stems from its cereal foodplants, such as *Polygonum* (knotweeds) and *Rumex* (docks). On account of this, *G. polygoni* represents a potentially important biocontrol agent of these cereals which can behave as weeds in agricultural settings ( Lesage & Majka 2009). For example, biocontrol of the knotweed *Polygonum aviculare* by *G. polygoni* has been proposed for Italian alfalfa farms ( Marrocchi, 1994). Furthermore, significant damage to wild buckwheat was noted in the 1950s in Saskatchewan ( Handford & Arrand, 1958). *G. polygoni* could pose an economic threat where buckwheat is itself cultivated, but empirical evidence so far suggests that this threat is low (e.g. Lühmann, 1938). However, *G. polygoni* also threatens rhubarb ( Balachowsky & Mesnil, 1936).

The genome of *Gastrophysa polygoni* was sequenced as part of the Darwin Tree of Life Project, a collaborative effort to sequence all named eukaryotic species in the Atlantic Archipelago of Britain and Ireland. This genome sequence may contribute to the verification of earlier cytological studies of chrysomelid karyotypes ( Petitpierre, 1978), which found that the modal number of the Chrysomelinae is 2 *n* = 24. Additionally, the genome sequence could aid our understanding of the molecular mechanisms employed by *G. polygoni* as a biocontrol agent and agricultural pest, as well as predict and address pesticide resistance in this species. Furthermore, the family Chrysomelidae contains many other economically important species, and this assembly will facilitate the study of the group as a whole.

## Genome sequence report

The genome of *Gastrophysa polygoni* ( Figure 1) was sequenced using Pacific Biosciences single-molecule HiFi long reads, generating a total of 32.10 Gb (gigabases) from 2.68 million reads, providing an estimated 79-fold coverage of the genome, based on the genome size estimate calculated in GenomeScope. Primary assembly contigs were scaffolded with chromosome conformation Hi-C data, which produced 116.78 Gb from 773.40 million reads. Specimen and sequencing details are provided in Table 1.

**Table 1.** Specimen and sequencing data for *Gastrophysa polygoni*.

Project information			
**Study title**	*Gastrophysa polygoni* (knotweed leaf beetle)		
**Umbrella BioProject**	PRJEB64716		
**Species**	*Gastrophysa polygoni*		
**BioSample**	SAMEA112232774		
**NCBI taxonomy ID**	878018		
Specimen information			
**Technology**	**ToLID**	**BioSample accession**	**Organism part**
**PacBio long read sequencing**	icGasPoly1	SAMEA112233274	Whole organism
**Hi-C sequencing**	icGasPoly1	SAMEA112233274	Whole organism
Sequencing information			
**Platform**	**Run accession**	**Read count**	**Base count (Gb)**
**Hi-C Illumina NovaSeq 6000**	ERR11814106	7.73e+08	116.78
**PacBio Sequel IIe**	ERR11809140	2.68e+06	32.1

Assembly errors were corrected by manual curation, including 25 missing joins or mis-joins and two haplotypic duplications. This reduced the assembly length by 0.49% and the scaffold number by 50.0%, and increased the scaffold N50 by 20.69%. The final assembly has a total length of 369.80 Mb in 19 sequence scaffolds, with 198 gaps, and a scaffold N50 of 30.5 Mb ( Table 2).

**Table 2.** Genome assembly data for *Gastrophysa polygoni*, icGasPoly1.1.

Genome assembly		
Assembly name	icGasPoly1.1	
Assembly accession	GCA_963576655.1	
*Accession of alternate haplotype*	*GCA_963576645.1*	
Span (Mb)	369.80	
Number of contigs	218	
Number of scaffolds	19	
Longest scaffold (Mb)	54.23	
Assembly metrics *		*Benchmark*
Contig N50 length (Mb)	2.8	*≥ 1 Mb*
Scaffold N50 length (Mb)	30.5	*= chromosome N50*
Consensus quality (QV)	61.0	*≥ 40*
*k*-mer completeness	100.0%	*≥ 95%*
BUSCO **	C:98.5%[S:97.7%,D:0.8%], F:0.3%,M:1.2%,n:2,124	*S > 90%* *D < 5%*
Percentage of assembly mapped to chromosomes	99.89%	*≥ 90%*
Sex chromosomes	X	*localised homologous pairs*
Organelles	Mitochondrial genome: 17.11 kb	*complete single alleles*

* Assembly metric benchmarks are adapted from Rhie *et al.* (2021) and the Earth BioGenome Project Report on Assembly Standards September 2024. ** BUSCO scores based on the endopterygota_odb10 BUSCO set using version 5.4.3. C = complete [S = single copy, D = duplicated], F = fragmented, M = missing, n = number of orthologues in comparison.

**Figure 2.**
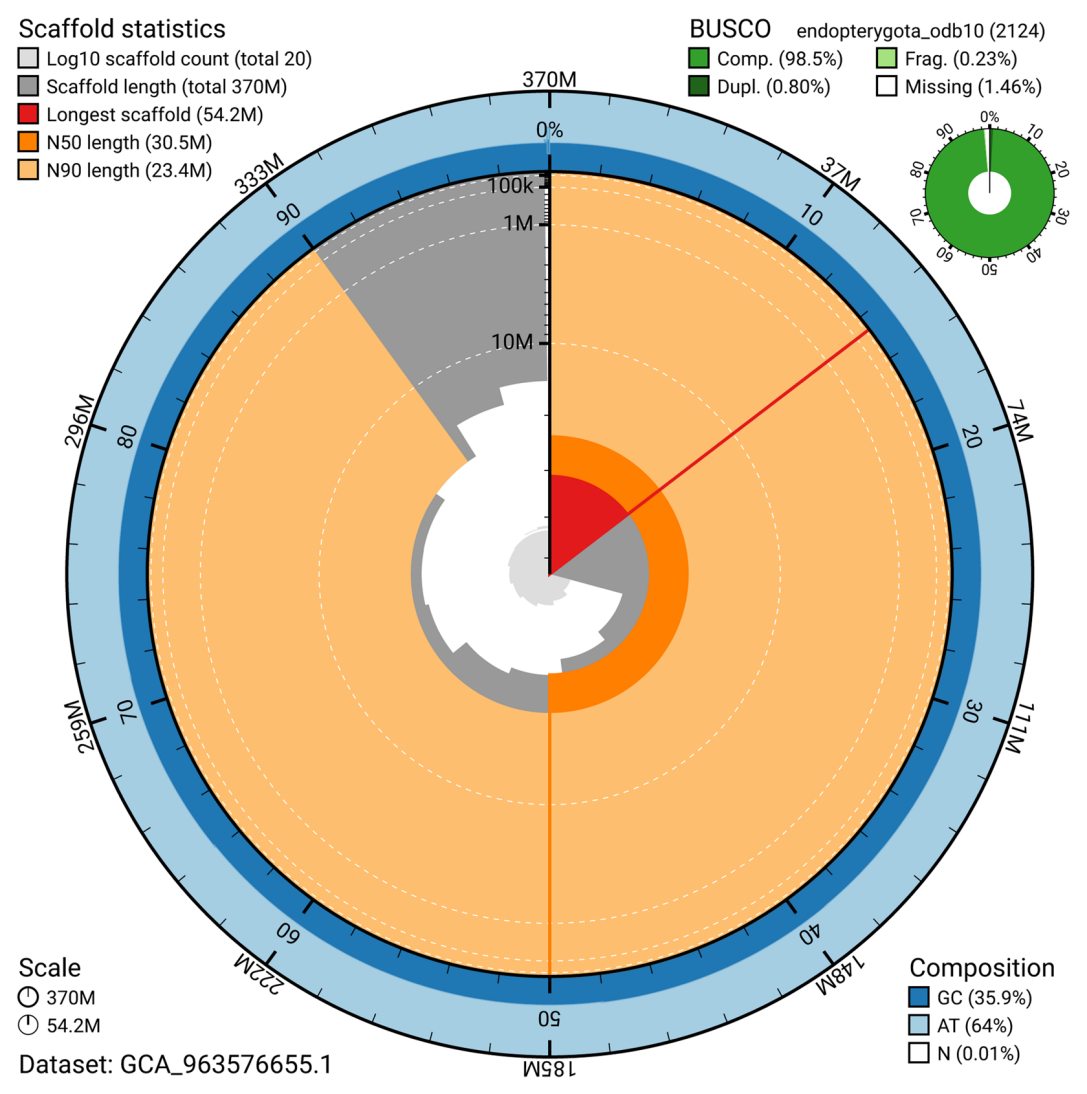
Genome assembly of *Gastrophysa polygoni*, icGasPoly1.1: metrics. The BlobToolKit snail plot shows N50 metrics and BUSCO gene completeness. The main plot is divided into 1,000 size-ordered bins around the circumference with each bin representing 0.1% of the 369,770,545 bp assembly. The distribution of scaffold lengths is shown in dark grey with the plot radius scaled to the longest scaffold present in the assembly (54,234,254 bp, shown in red). Orange and pale-orange arcs show the N50 and N90 scaffold lengths (30,539,640 and 23,360,099 bp), respectively. The pale grey spiral shows the cumulative scaffold count on a log scale with white scale lines showing successive orders of magnitude. The blue and pale-blue area around the outside of the plot shows the distribution of GC, AT and N percentages in the same bins as the inner plot. A summary of complete, fragmented, duplicated and missing BUSCO genes in the endopterygota_odb10 set is shown in the top right. An interactive version of this figure is available at https://blobtoolkit.genomehubs.org/view/GCA_963576655.1/dataset/GCA_963576655.1/snail.

**Figure 3.**
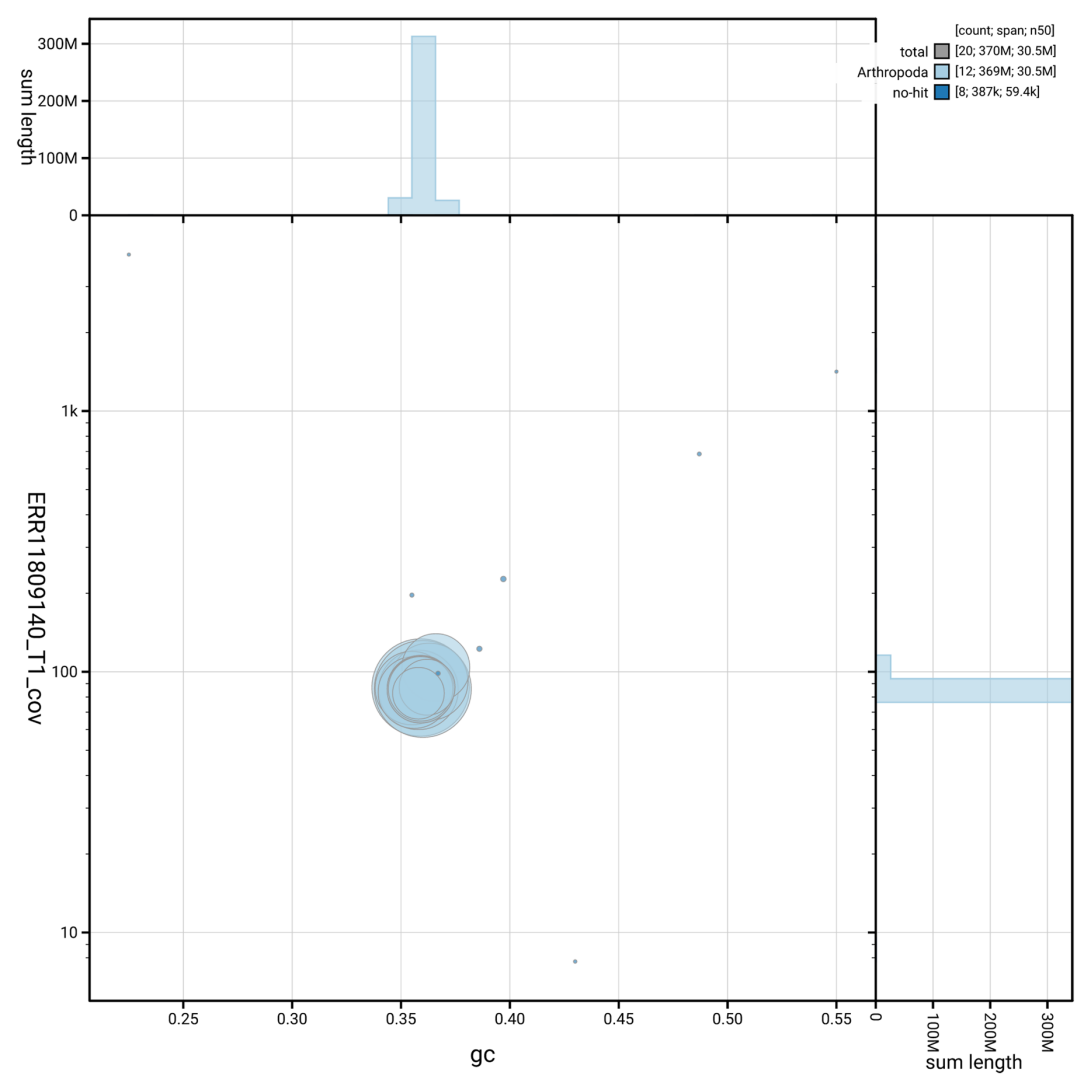
Genome assembly of *Gastrophysa polygoni*, icGasPoly1.1: BlobToolKit GC-coverage plot showing sequence coverage (vertical axis) and GC content (horizontal axis). The circles represent scaffolds, with the size proportional to scaffold length and the colour representing phylum membership. The histograms along the axes display the total length of sequences distributed across different levels of coverage and GC content. An interactive version of this figure is available at https://blobtoolkit.genomehubs.org/view/GCA_963576655.1/dataset/GCA_963576655.1/blob.

**Figure 4.**
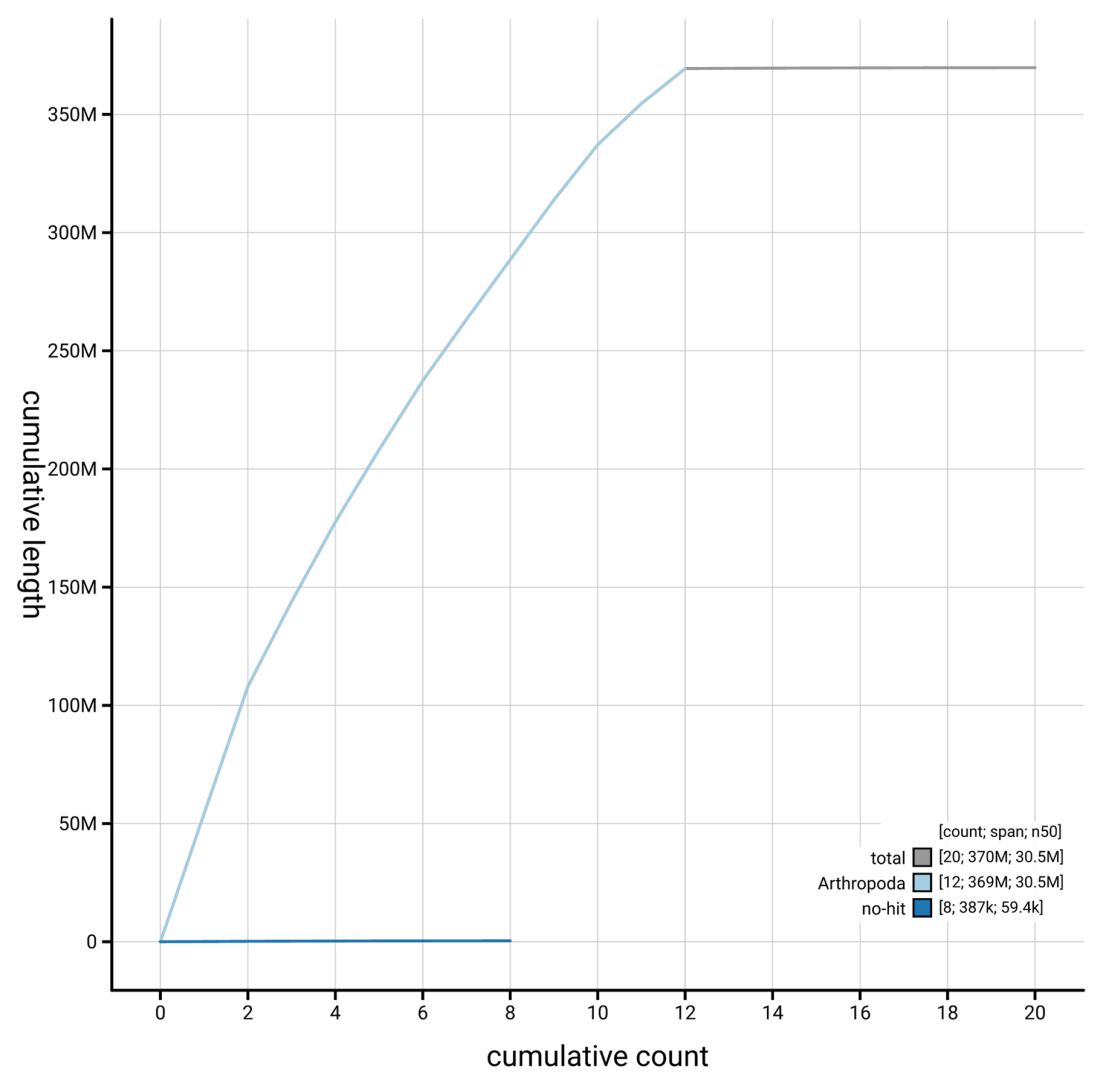
Genome assembly of *Gastrophysa polygoni* icGasPoly1.1: BlobToolKit cumulative sequence plot. The grey line shows cumulative length for all scaffolds. Coloured lines show cumulative lengths of scaffolds assigned to each phylum using the buscogenes taxrule. An interactive version of this figure is available at https://blobtoolkit.genomehubs.org/view/GCA_963576655.1/dataset/GCA_963576655.1/cumulative.

The snail plot in Figure 2 provides a summary of the assembly statistics, indicating the distribution of scaffold lengths and other assembly metrics. Figure 3 shows the distribution of scaffolds by GC proportion and coverage, helping to distinguish between various genomic elements. Figure 4 presents a cumulative assembly plot, with separate curves representing different scaffold subsets assigned to various phyla, illustrating the completeness of the assembly.

**Figure 5.**
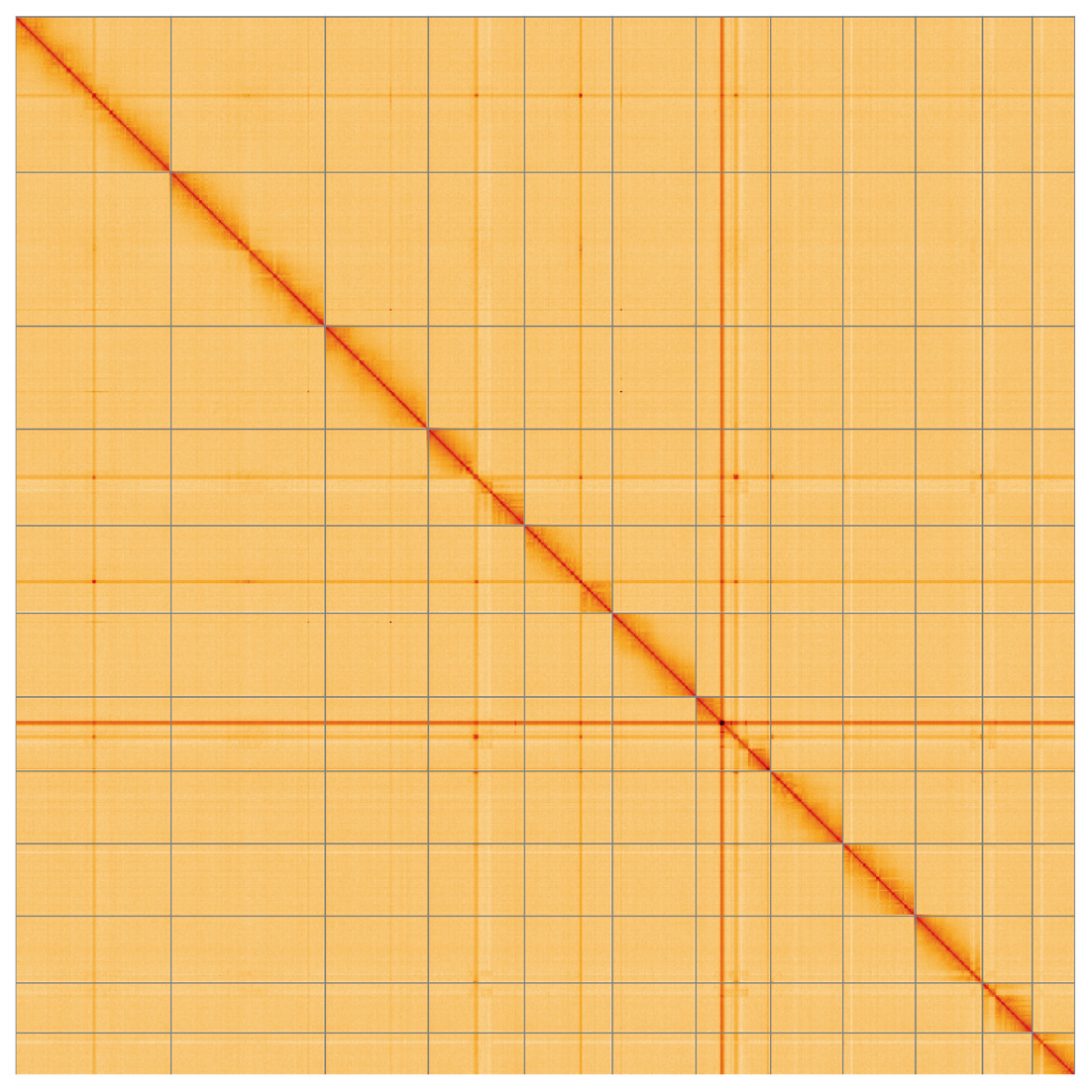
Genome assembly of *Gastrophysa polygoni* icGasPoly1.1: Hi-C contact map of the icGasPoly1.1 assembly, visualised using HiGlass. Chromosomes are shown in order of size from left to right and top to bottom. An interactive version of this figure may be viewed at https://genome-note-higlass.tol.sanger.ac.uk/l/?d=WK47rmOzT5-XqUbPUuRviA.

Most (99.89%) of the assembly sequence was assigned to 12 chromosomal-level scaffolds, representing 11 autosomes and the X sex chromosome. These chromosome-level scaffolds, confirmed by the Hi-C data, are named in order of size ( Figure 5; Table 3). During manual curation, the X chromosome was identified based on synteny with the *Chrysolina americana* assembly (GCA_958502065.1).

**Table 3.** Chromosomal pseudomolecules in the genome assembly of *Gastrophysa polygoni*, icGasPoly1.

INSDC accession	Name	Length (Mb)	GC%
OY755082.1	1	54.23	36.0
OY755083.1	2	53.75	36.0
OY755084.1	3	35.89	36.0
OY755086.1	4	30.54	35.5
OY755087.1	5	29.23	35.5
OY755088.1	6	25.93	36.5
OY755089.1	7	25.3	36.0
OY755090.1	8	25.26	36.0
OY755091.1	9	23.36	36.0
OY755092.1	10	17.4	36.0
OY755093.1	11	14.8	36.0
OY755085.1	X	33.69	36.5
OY755094.1	MT	0.02	23.0

The deposited assembly represents one haplotype. Contigs that correspond to an alternate haplotype have also been deposited. The mitochondrial genome was assembled and is provided as a contig within the multifasta file in the genome submission, and as a separate fasta file with accession OY755094.1.

The final assembly has a Quality Value (QV) of 61.0 and *k*-mer completeness of 100.0%. BUSCO (v5.4.3) analysis using the endopterygota_odb10 reference set ( *n* = 2,124) indicated a completeness score of 98.5% (single = 97.7%, duplicated = 0.8%).

Metadata for specimens, BOLD barcode results, spectra estimates, sequencing runs, contaminants and pre-curation assembly statistics are given at https://links.tol.sanger.ac.uk/species/878018.

## Methods

### Sample acquisition and DNA barcoding

An adult female specimen of *Gastrophysa polygoni* (specimen ID Ox002587, ToLID icGasPoly1) was collected from Wytham Woods, Oxfordshire, United Kingdom (latitude 51.77, longitude –1.34) on 2022-07-29 by potting. The specimen was collected and identified by James McCulloch (University of Oxford) and preserved on dry ice.

The initial identification was verified by an additional DNA barcoding process according to the framework developed by Twyford *et al*. (2024). A small sample was dissected from the specimens and stored in ethanol, while the remaining parts were shipped on dry ice to the Wellcome Sanger Institute (WSI). The tissue was lysed, the COI marker region was amplified by PCR, and amplicons were sequenced and compared to the BOLD database, confirming the species identification ( Crowley *et al.*, 2023). Following whole genome sequence generation, the relevant DNA barcode region was also used alongside the initial barcoding data for sample tracking at the WSI ( Twyford *et al.*, 2024). The standard operating procedures for Darwin Tree of Life barcoding have been deposited on protocols.io ( Beasley *et al.*, 2023).

### Nucleic acid extraction

The workflow for high molecular weight (HMW) DNA extraction at the Wellcome Sanger Institute (WSI) Tree of Life Core Laboratory includes a sequence of core procedures: sample preparation and homogenisation, DNA extraction, fragmentation and purification. Detailed protocols are available on protocols.io ( Denton *et al.*, 2023b). The icGasPoly1 sample was prepared for DNA extraction by weighing and dissecting it on dry ice ( Jay *et al.*, 2023), and tissue from the whole organism was homogenised using a PowerMasher II tissue disruptor ( Denton *et al.*, 2023a).

HMW DNA was extracted in the WSI Scientific Operations core using the Automated MagAttract v2 protocol ( Oatley *et al.*, 2023). The DNA was sheared into an average fragment size of 12–20 kb in a Megaruptor 3 system ( Bates *et al.*, 2023). Sheared DNA was purified by solid-phase reversible immobilisation, using AMPure PB beads to eliminate shorter fragments and concentrate the DNA ( Strickland *et al.*, 2023). The concentration of the sheared and purified DNA was assessed using a Nanodrop spectrophotometer and Qubit Fluorometer using the Qubit dsDNA High Sensitivity Assay kit. Fragment size distribution was evaluated by running the sample on the FemtoPulse system.

### Hi-C preparation

Tissue from the icGasPoly1 sample (whole organism) was processed at the WSI Scientific Operations core, using the Arima-HiC v2 kit. In brief, frozen tissue (stored at –80°C) was fixed, and the DNA crosslinked using a TC buffer with 22% formaldehyde. After crosslinking, the tissue was homogenised using the Diagnocine Power Masher-II and BioMasher-II tubes and pestles. Following the kit manufacturer's instructions, crosslinked DNA was digested using a restriction enzyme master mix. The 5’-overhangs were then filled in and labelled with biotinylated nucleotides and proximally ligated. An overnight incubation was carried out for enzymes to digest remaining proteins and for crosslinks to reverse. A clean up was performed with SPRIselect beads prior to library preparation.

### Library preparation and sequencing

Library preparation and sequencing were performed at the WSI Scientific Operations core. Pacific Biosciences HiFi circular consensus DNA sequencing libraries were prepared using the PacBio Express Template Preparation Kit v2.0 (Pacific Biosciences, California, USA) as per the manufacturer's instructions. The kit includes the reagents required for removal of single-strand overhangs, DNA damage repair, end repair/A-tailing, adapter ligation, and nuclease treatment. Library preparation also included a library purification step using AMPure PB beads (Pacific Biosciences, California, USA) and size selection step to remove templates <3kb using AMPure PB modified SPRI. DNA concentration was quantified using the Qubit Fluorometer v2.0 and Qubit HS Assay Kit and the final library fragment size analysis was carried out using the Agilent Femto Pulse Automated Pulsed Field CE Instrument and gDNA 165 kb gDNA and 55 kb BAC analysis kit. Samples were sequenced using the Sequel IIe system (Pacific Biosciences, California, USA). The concentration of the library loaded onto the Sequel IIe was between 40–135 pM. The SMRT link software, a PacBio web-based end-to-end workflow manager, was used to set-up and monitor the run, as well as perform primary and secondary analysis of the data upon completion.

For Hi-C library preparation, DNA was fragmented to a size of 400 to 600 bp using a Covaris E220 sonicator. The DNA was then enriched, barcoded, and amplified using the NEBNext Ultra II DNA Library Prep Kit following manufacturers’ instructions. The Hi-C sequencing was performed using paired-end sequencing with a read length of 150 bp on an Illumina NovaSeq 6000 instrument.

### Genome assembly, curation and evaluation

***Assembly***

The HiFi reads were first assembled using Hifiasm ( Cheng *et al.*, 2021) with the --primary option. Haplotypic duplications were identified and removed using purge_dups ( Guan *et al.*, 2020). The Hi-C reads were mapped to the primary contigs using bwa-mem2 ( Vasimuddin *et al.*, 2019). The contigs were further scaffolded using the provided Hi-C data ( Rao *et al.*, 2014) in YaHS ( Zhou *et al.*, 2023) using the --break option. The scaffolded assemblies were evaluated using Gfastats ( Formenti *et al.*, 2022), BUSCO ( Manni *et al.*, 2021) and MERQURY.FK ( Rhie *et al.*, 2020).

The mitochondrial genome was assembled using MitoHiFi ( Uliano-Silva *et al.*, 2023), which runs MitoFinder ( Allio *et al.*, 2020) and uses these annotations to select the final mitochondrial contig and to ensure the general quality of the sequence.

***Assembly curation***

The assembly was decontaminated using the Assembly Screen for Cobionts and Contaminants (ASCC) pipeline (article in preparation). Flat files and maps used in curation were generated in TreeVal ( Pointon *et al.*, 2023). Manual curation was primarily conducted using PretextView ( Harry, 2022), with additional insights provided by JBrowse2 ( Diesh *et al.*, 2023) and HiGlass ( Kerpedjiev *et al.*, 2018). Scaffolds were visually inspected and corrected as described by Howe *et al*. (2021). Any identified contamination, missed joins, and mis-joins were corrected, and duplicate sequences were tagged and removed. Synteny analysis was performed to identify the X chromosome. The curation process is documented at https://gitlab.com/wtsi-grit/rapid-curation (article in preparation).

***Evaluation of the final assembly***

The final assembly was post-processed and evaluated using the three Nextflow ( Di Tommaso *et al.*, 2017) DSL2 pipelines: sanger-tol/readmapping ( Surana *et al.*, 2023a), sanger-tol/genomenote ( Surana *et al.*, 2023b), and sanger-tol/blobtoolkit ( Muffato *et al.*, 2024). The readmapping pipeline aligns the Hi-C reads using bwa-mem2 ( Vasimuddin *et al.*, 2019) and combines the alignment files with SAMtools ( Danecek *et al.*, 2021). The genomenote pipeline converts the Hi-C alignments into a contact map using BEDTools ( Quinlan & Hall, 2010) and the Cooler tool suite ( Abdennur & Mirny, 2020). The contact map is visualised in HiGlass ( Kerpedjiev *et al.*, 2018). This pipeline also generates assembly statistics using the NCBI datasets report ( Sayers *et al.*, 2024), computes *k*-mer completeness and QV consensus quality values with FastK and MERQURY.FK, and runs BUSCO ( Manni *et al.*, 2021) to assess completeness.

The blobtoolkit pipeline is a Nextflow port of the previous Snakemake Blobtoolkit pipeline ( Challis *et al.*, 2020). This pipeline aligns the PacBio reads in SAMtools and minimap2 ( Li, 2018) and generates coverage tracks for regions of fixed size. In parallel, it queries the GoaT database ( Challis *et al.*, 2023) to identify all matching BUSCO lineages to run BUSCO ( Manni *et al.*, 2021). For the three domain-level BUSCO lineages, the pipeline aligns the BUSCO genes to the UniProt Reference Proteomes database ( Bateman *et al.*, 2023) with DIAMOND blastp ( Buchfink *et al.*, 2021). The genome is also split into chunks according to the density of the BUSCO genes from the closest taxonomic lineage, and each chunk is aligned to the UniProt Reference Proteomes database with DIAMOND blastx. Genome sequences without a hit are chunked with seqtk and aligned to the NT database with blastn ( Altschul *et al.*, 1990). The blobtools suite combines all these outputs into a blobdir for visualisation.

The genome assembly and evaluation pipelines were developed using nf-core tooling ( Ewels *et al.*, 2020) and MultiQC ( Ewels *et al.*, 2016), relying on the Conda package manager, the Bioconda initiative ( Grüning *et al.*, 2018), the Biocontainers infrastructure ( da Veiga Leprevost *et al.*, 2017), as well as the Docker ( Merkel, 2014) and Singularity ( Kurtzer *et al.*, 2017) containerisation solutions.

Table 4 contains a list of relevant software tool versions and sources.

**Table 4.** Software tools: versions and sources.

Software tool	Version	Source
BEDTools	2.30.0	https://github.com/arq5x/bedtools2
BLAST	2.14.0	ftp://ftp.ncbi.nlm.nih.gov/blast/executables/blast+/
BlobToolKit	4.3.7	https://github.com/blobtoolkit/blobtoolkit
BUSCO	5.4.3 and 5.5.0	https://gitlab.com/ezlab/busco
bwa-mem2	2.2.1	https://github.com/bwa-mem2/bwa-mem2
Cooler	0.8.11	https://github.com/open2c/cooler
DIAMOND	2.1.8	https://github.com/bbuchfink/diamond
fasta_windows	0.2.4	https://github.com/tolkit/fasta_windows
FastK	427104ea91c78c3b8b8b49f1a7d6bbeaa869ba1c	https://github.com/thegenemyers/FASTK
Gfastats	1.3.6	https://github.com/vgl-hub/gfastats
GoaT CLI	0.2.5	https://github.com/genomehubs/goat-cli
Hifiasm	0.19.8-r587	https://github.com/chhylp123/hifiasm
HiGlass	44086069ee7d4d3f6f3f0012569789ec138f42b84a a44357826c0b6753eb28de	https://github.com/higlass/higlass
Merqury.FK	d00d98157618f4e8d1a9190026b19b471055b22e	https://github.com/thegenemyers/MERQURY.FK
MitoHiFi	3	https://github.com/marcelauliano/MitoHiFi
MultiQC	1.14, 1.17, and 1.18	https://github.com/MultiQC/MultiQC
NCBI Datasets	15.12.0	https://github.com/ncbi/datasets
Nextflow	23.04.0-5857	https://github.com/nextflow-io/nextflow
PretextView	0.2	https://github.com/sanger-tol/PretextView
purge_dups	1.2.5	https://github.com/dfguan/purge_dups
samtools	1.16.1, 1.17, and 1.18	https://github.com/samtools/samtools
sanger-tol/ascc	-	https://github.com/sanger-tol/ascc
sanger-tol/genomenote	1.1.1	https://github.com/sanger-tol/genomenote
sanger-tol/readmapping	1.2.1	https://github.com/sanger-tol/readmapping
Seqtk	1.3	https://github.com/lh3/seqtk
Singularity	3.9.0	https://github.com/sylabs/singularity
TreeVal	1.0.0	https://github.com/sanger-tol/treeval
YaHS	1.2a.2	https://github.com/c-zhou/yahs

### Wellcome Sanger Institute – Legal and Governance

The materials that have contributed to this genome note have been supplied by a Darwin Tree of Life Partner. The submission of materials by a Darwin Tree of Life Partner is subject to the **‘Darwin Tree of Life Project Sampling Code of Practice’**, which can be found in full on the Darwin Tree of Life website here. By agreeing with and signing up to the Sampling Code of Practice, the Darwin Tree of Life Partner agrees they will meet the legal and ethical requirements and standards set out within this document in respect of all samples acquired for, and supplied to, the Darwin Tree of Life Project.

Further, the Wellcome Sanger Institute employs a process whereby due diligence is carried out proportionate to the nature of the materials themselves, and the circumstances under which they have been/are to be collected and provided for use. The purpose of this is to address and mitigate any potential legal and/or ethical implications of receipt and use of the materials as part of the research project, and to ensure that in doing so we align with best practice wherever possible. The overarching areas of consideration are:

•   Ethical review of provenance and sourcing of the material

•   Legality of collection, transfer and use (national and international)

Each transfer of samples is further undertaken according to a Research Collaboration Agreement or Material Transfer Agreement entered into by the Darwin Tree of Life Partner, Genome Research Limited (operating as the Wellcome Sanger Institute), and in some circumstances other Darwin Tree of Life collaborators.

## Data Availability

European Nucleotide Archive: *Gastrophysa polygoni* (knotweed leaf beetle). Accession number PRJEB64716; https://identifiers.org/ena.embl/PRJEB64716. The genome sequence is released openly for reuse. The *Gastrophysa polygoni* genome sequencing initiative is part of the Darwin Tree of Life (DToL) project. All raw sequence data and the assembly have been deposited in INSDC databases. The genome will be annotated using available RNA-Seq data and presented through the Ensembl pipeline at the European Bioinformatics Institute. Raw data and assembly accession identifiers are reported in Table 1 and Table 2.

## Author information

Members of the University of Oxford and Wytham Woods Genome Acquisition Lab are listed here: https://doi.org/10.5281/zenodo.12157525.

Members of the Darwin Tree of Life Barcoding collective are listed here: https://doi.org/10.5281/zenodo.12158331.

Members of the Wellcome Sanger Institute Tree of Life Management, Samples and Laboratory team are listed here: https://doi.org/10.5281/zenodo.12162482.

Members of Wellcome Sanger Institute Scientific Operations: Sequencing Operations are listed here: https://doi.org/10.5281/zenodo.12165051.

Members of the Wellcome Sanger Institute Tree of Life Core Informatics team are listed here: https://doi.org/10.5281/zenodo.12160324.

Members of the Tree of Life Core Informatics collective are listed here: https://doi.org/10.5281/zenodo.12205391.

Members of the Darwin Tree of Life Consortium are listed here: https://doi.org/10.5281/zenodo.4783558.
