# Supplementary material for: The genome sequence of knotweed leaf beetle, Gastrophysa polygoni (Linnaeus, 1758)
Source: Wellcome Open Res. 2024 Nov 4;9:642. [Version 1] doi: 10.12688/wellcomeopenres.23293.1 (PMC11617825; doi:10.12688/wellcomeopenres.23293.1)
Supplement: Peer review history

# Reviewer report: Reviewer response for version 1

**DOI:** 10.21956/wellcomeopenres.25675.r112284

**Published:** 2024-12-04

**Authors:** Na Ra Shin

**Affiliations:** 1 The University of Memphis, Memphis, Tennessee, USA

This manuscript presents a high-quality genome assembly for the knotweed leaf beetle, supported by comprehensive background information about the species. The authors have also included details and links to the tools and methods used, facilitating reproducibility and enabling others to replicate their analyses. This dataset will be valuable to researchers interested in this species.

 Minor suggestion:

 1. Include the family and order in the title for clarity and broader relevance.

Are sufficient details of methods and materials provided to allow replication by others?

Partly

Is the rationale for creating the dataset(s) clearly described?

Yes

Are the datasets clearly presented in a useable and accessible format?

Yes

Are the protocols appropriate and is the work technically sound?

Yes

Reviewer Expertise:

Insect genome and transcriptome, Beetles evolution, Systematic, Metabolic evolution

I confirm that I have read this submission and believe that I have an appropriate level of expertise to confirm that it is of an acceptable scientific standard.

# Reviewer report: Reviewer response for version 1

**DOI:** 10.21956/wellcomeopenres.25675.r112288

**Published:** 2024-12-02

**Authors:** James RM Bickerstaff

**Affiliations:** 1 CSIRO Consortium, Canberra, Australian Capital Territory, Australia

The authors present a high quality genome for the knotweed leaf beetle with good sequence statistics. The protocols presented herein are appropriate for the objective of genome assembly and sound, and evidently have led to a good genome assembly. My only concern is the brevity in which details of the bioinformatic pipelines are presented. I think some more detail should be included so that your pipelines are fully repeatable. Primarily, I have three questions regarding the methods used:

 1) why was the --primary flag invoked for the assembly, did you not want to fully phase the alternate assembly as well? 

 For the Hi-C scaffolding, did you fully use the Arima mapping pipeline? Arima Hi-C scaffolding requires several further steps for curation and data cleaning as presented in their pipeline.

 How was the synteny analysis completed? What species did you compare with, and did you compare with more than one species?

Are sufficient details of methods and materials provided to allow replication by others?

Partly

Is the rationale for creating the dataset(s) clearly described?

Yes

Are the datasets clearly presented in a useable and accessible format?

Yes

Are the protocols appropriate and is the work technically sound?

Yes

Reviewer Expertise:

Entomology, genomics, population genetics, phylogenetics

I confirm that I have read this submission and believe that I have an appropriate level of expertise to confirm that it is of an acceptable scientific standard.
